# *In vitro* cytokine response of circulating mononuclear cells from healthy dogs to stage-specific antigens of *Angiostrongylus vasorum*

**DOI:** 10.1186/s12917-025-04977-5

**Published:** 2025-10-02

**Authors:** Janine Hertaeg, Ulisse Salazar, Johannes vom Berg, Salomé LeibundGut-Landmann, Andreas W. Oehm, Manuela Schnyder

**Affiliations:** 1 Institute of Parasitology, University of Zurich, Zurich, Switzerland; 2 Institute of Laboratory Animal Science, University of Zurich, Schlieren, Switzerland; 3 Section of Immunology at Vetsuisse Faculty, University of Zurich, Zurich, Switzerland; 4 Institute of Experimental Immunology, University of Zurich, Zurich, Switzerland; 5 Medical Research Council Centre for Medical Mycology at the University of Exeter, Department of Biosciences, Faculty of Health and Life Sciences, Exeter, UK; 6 Graduate School for Cellular and Biomedical Sciences (GCB), University of Bern, Bern, Switzerland

**Keywords:** Canine angiostronylosis, Immunobiology, Immunomodulation, Host-helminth interaction

## Abstract

**Background:**

Circulating lymphocytes are essential for the immune response to helminth infections, as they secrete cytokines and thus coordinate subsequent immunological processes. In canine angiostrongylosis, a potentially fatal disease caused by the metastrongylid nematode *Angiostrongylus vasorum*, the underlying immune mechanisms driving disease progression remain poorly understood despite the severe clinical consequences. Canine peripheral blood mononuclear cells (PBMCs) were isolated from healthy dogs and stimulated with three different antigens of *A vasorum*, including adult excretory-secretory products (ESP), adult full-worm somatic antigen, and first-stage larval (L1) somatic antigen. Temporal dynamics and magnitude of relative cytokine expression (IFNγ, TNF, IL-4, IL-6, IL-10, IL-13) was evaluated after 4 h, 24 h, and 5 days of antigen exposure via quantitative reverse transcription PCR. To determine whether the cytokine expression changes translated into shifts in circulating mononuclear cell subsets or proliferative activity, phenotypic characterisation by flow cytometry was conducted after a 72 h stimulation with L1 antigen or ESP, and compared to unstimulated controls.

**Results:**

Early responses varied across antigen types, with ESP promoting a regulatory cytokine profile with modest upregulation of IL-4, IL-6, and IL-10, and downregulation of IFNγ, TNF, and IL-13. Adult antigen induced increased expression of all examined cytokines, while L1 antigen triggered the strongest inflammatory response compared to the other antigens. At 24 h, all responses were amplified, particularly those to L1 and adult antigen, and showed a shift towards a Th2 cytokine profile with increased IL-4 and IL-13 expression. By five days, IL-4 and IL-13 remained predominant. No change in the relative abundance of major immune cell populations (CD4⁺ T cells, CD8⁺ T cells, B cells, neutrophils, monocytes, and CD14⁻ CD22⁻ antigen-presenting cells) was observed in flow cytometry following stimulation. However, a notable increase in Ki67 expression, a marker of cell proliferation, was detected in CD8⁺ T cells after L1 antigen stimulation.

**Conclusions:**

Distinct cytokine profiles elicited by different *A. vasorum* antigens suggest that the parasite’s modulation of host immunity and induction of stage-specific responses are key to persistence and clinical presentation of canine angiostrongylosis. Further investigation into the antigenic components and immune pathways may lead to tailored therapies, improved clinical management, and to a deeper understanding of stage-specific aspects of helminth infections.

## Introduction

Helminth infections present a substantial challenge to the host immune system, demanding a well-coordinated response to effectively counter the parasitic invaders. These infections initiate a complex interaction between innate and adaptive immunity, with peripheral blood mononuclear cells (PBMCs)—including lymphocytes (T cells, B cells) and monocytes—playing a pivotal role [1–3]. T cells, particularly CD4 + T cells, differentiate into Th2 cells producing key cytokines such as interleukin (IL-)4 and IL-13. These cytokines play a crucial role in orchestrating the immune response to helminths by promoting antibody production and activating effector cells. Among other cytokines involved in anti-helminth immunity, IL-5 is essential for eosinophil activation and recruitment. IL-10 plays an immunoregulatory role, helping to balance inflammation and prevent excessive tissue damage. Interferon-gamma (IFN-γ) primarily produced by Th1 cells, counteracts Th2 response and is involved in macrophage activation. Tumor necrosis factor (TNF) and IL-6 contribute to inflammatory signalling and immune cell recruitment, although their roles in helminth infection can be context-dependent. Regulatory T cells (Tregs) and alternatively activated macrophages, instructed by IL-10 and also themselves producing IL-10, help to balance this response, preventing excessive inflammation and tissue damage [1–7].

Helminths actively disrupt the balance between Th2 and Th1 immune responses and induce immunosuppressive cytokines like IL-10, dampening pro-inflammatory responses and facilitating parasite persistence. Through these mechanisms, helminths manipulate host immunity, evade clearance, and establish chronic infections [1–5]. Helminths possess a highly effective excretory/secretory (ES) system, which not only facilitates tissue invasion and a state of tolerance towards the parasite but also promotes wound healing, tissue remodeling, and dampening of or evasion from the inflammatory response [4, 6–9]. These mechanisms include inhibiting immune cell signaling, blocking the migration of antigen-presenting cells (APCs), and secreting proteases that degrade antibodies designed to trap the parasites [4–10].

An important hallmark of helminth immune evasion is the stage-specific expression of antigens. Helminths express different sets of antigens during various life cycle stages, which can differentially affect the host’s immune response. During the early larval stage, helminths often produce antigens that help them evade initial immune defenses, facilitating their migration through tissues, and establishment in host tissues. In contrast, adult-stage antigens are involved in nutrient acquisition and long-term helminth survival, thereby further modulating the immune system to ensure persistence. The differential antigen expression at different stages of the helminth life cycle helps the parasite to avoid immune detection and destruction, contributing to chronic infection.

*Angiostrongylus vasorum* is a metastrongylid nematode parasite that primarily infects the pulmonary arteries and right heart of canids, especially foxes and domestic dogs. Infection with this parasite can result in a severe, potentially fatal disease characterised by respiratory distress, coagulopathies, neurological disorders, and cardiac dysfunction [11–13]. Infected dogs exhibit high and persisting levels of serum antibodies directed against *A. vasorum*, yet these do not provide protection against reinfection nor do they guarantee the clearance of current infections [14, 15]. During *A. vasorum* infection, circulating mononuclear cells may encounter parasite antigens through multiple routes. Adult worms reside in blood vessels and release excretory-secretory products (ESP) that enter the circulation. First-stage larvae (L1) may transiently appear in pulmonary capillaries before migrating into lung tissue, potentially liberating antigens into the blood. Moreover, antigenic material released during tissue migration of L1 may also reach circulating immune cells [16–19]. Currently, little is known of the immune response to *A. vasorum* infection in dogs, including which antigens are responsible for initiating an immune response, facilitate host immune evasion, or contribute to pathogenesis. Studying how specific antigenic components of the parasite, like ESP and stage-specific antigens, modulate the immune response can lead to more targeted approaches for controlling helminth infections [1, 10, 20]. For instance, stage-specific antigens might be exploited to develop vaccines that elicit protective responses against migratory larvae, diagnostic assays that detect stage-specific immune signatures for early detection or prognostic stratification, adjuvant selection to steer cytokine profiles toward beneficial responses, and immunomodulatory therapies that limit pathological inflammation while supporting parasite clearance.

## Methods

In this study, we aimed at (I) investigating the kinetics and magnitude of cytokine profiles (Th1 vs. Th2 vs. regulatory) in canine circulating mononuclear cells following exposure to three distinct antigens of *A. vasorum* (adult ESP, adult full somatic antigen, and L1 full somatic antigen) which are considered essential in host-parasite interaction and may be involved in immune activation or evasion [1, 4, 5, 20, 21]. Moreover, we set out to (II) determine whether the transcriptional changes are accompanied by changes in immune cell phenotypes or profileration, focussing on two antigens (ESP and L1 antigen) for flow cytometric analysis at a 72 h time point.

### Blood sampling

Blood samples were taken from three healthy female and four male beagle dogs owned by the University of Zurich. These dogs were clinically healthy, without any signs of disease, and had no infection with *Angiostrongylus vasorum* or other relevant pathogens, as confirmed by veterinary examination and diagnostic screening. They were maintained under standard husbandry conditions and had not received any immunomodulatory treatments, vaccinations, or anti-parasitic medications within at least 4–6 weeks prior to sampling, ensuring an unaltered baseline immune status before sample collection. Their ages ranged from three to seven years. Whole blood was collected by jugular venipuncture using 20G VACUETTE^R^ Multiple Use Drawing Needles (Greiner Bio-One, Kremsmünster, Austria) into 9 ml EDTA-treated VACUETTE^R^ tubes (Greiner Bio-One, Kremsmünster, Austria). For venipuncture, dogs were restrained by an assistant, and blood was drawn swiftly without anaesthesia according to good veterinary practice.

### Isolation of peripheral blood mononuclear cells (PBMCs)

The isolation of PBMCs was initiated within two hours after blood sampling. PBMCs were isolated from whole blood by density gradient centrifugation using Histopaque − 1077 (Sigma Aldrich, Buchs, Switzerland), which enriches for mononuclear cells (lymphocytes and monocytes) while excluding granulocytes (including neutrophils) and erythrocytes. After PBMC isolation, an aliquot of cells was mixed 1:1 with 0.4% Trypan Blue solution and counted using a Neubauer chamber. Viability consistently exceeded 95%. Cells were cultured in RPMI 1640 medium (Sigma Aldrich, Buchs, Switzerland) supplemented with 10% ml foetal calf serum (Sigma Aldrich, Buchs Switzerland), 2% of 0.6 mg/ml of penicillin together with 10 mg/ml streptomycin (Sigma Aldrich, Buchs, Switzerland), 1% Minimum Essential Medium (MEM) Vitamin solution (Sigma-Aldrich, Buchs, Switzerland), 1% L-Glutamine (200mM; SigmaAldrich, Buchs, Switzerland), 2% sodium pyruvate (100mM, Sigma Aldrich, Sigma Aldrich, Buchs, Switzerland), and 1% MEM Non-essential Amino Acid Solution (100x; Sigma Aldrich, Buchs, Switzerland).

### Antigen preparation

*Angiostrongylus vasorum* ESP were obtained as previously described [22]. Full somatic antigen extracts were prepared from L1 and adult specimens of *A. vasorum* according to Oehm et al. [23]. Protein concentration of all antigens was determined using the Pierce™ BCA Protein Assay Kit (Thermo Fisher Scientific, Basel, Switzerland). Endotoxin levels were determined prior to the use for stimulation using the Pierce™ Chromogenic Endotoxin Quant Kit (Thermo Fisher Scientific, Basel Switzerland). Only antigens with an endotoxin concentration below 0.01 ng/ml were used for stimulation. To remove endotoxin from samples with an initially higher concentration, the Pierce™ High-Capacity Endotoxin Removal Resin (Thermo Fisher Scientific, Basel, Switzerland) was used.

### Stimulation of PBMCs and RT-qPCR

Cells were seeded at 500,000 cells per ml in each well containing pre-warmed cultivation medium in a 24-well cell culture plate. Cells were allowed to rest over night at 37°C and 5% CO_2_. The following day, the medium was replaced, and cells were stimulated with 10 µg *A. vasorum* ESP (from a 0.15 mg/ml stock solution), adult somatic antigen (from a 4.2 mg/ml stock solution), or L1 somatic antigen (from a 0.2 mg/ml stock solution), ensuring equal protein input across conditions. All stimulations were carried out in triplicates. As a positive control for cell viability and responsiveness, PBMCs were stimulated in parallel with 10 ng/well ionomycin and 100 ng/well phorbol-12-myristate-13-acetate (PMA) (both Sigma-Aldrich, Buchs, Switzerland). This served as an internal control to verify assay integrity but was not included in downstream analyses. Unstimulated PBMCs served as negative controls for each time point. For each donor and each time point, PBMCs were incubated without *A. vasorum* antigens. Instead, the negative control received an equivalent volume of sterile 1× phosphate-buffered saline (PBS), matching the buffer used to dissolve the antigens. To ensure specificity and exclude contamination, two types of negative controls were included in the RT-qPCR workflow. No-template controls (NTCs), in which water replaced the cDNA, were included to confirm the absence of contamination in reagents. Additionally, no-reverse transcriptase (no-RT) controls, in which the reverse transcriptase enzyme was omitted during cDNA synthesis, were used for each sample to verify that amplification originated exclusively from RNA rather than genomic DNA.

Total RNA was extracted using the RNeasy^®^ mini kit (Qiagen, Hombrechtikon, Switzerland) according to the manufacturer’s instructions at three different time points: four hours, 24 h, and five days after stimulation. A total amount of 250 ng of RNA was transcribed into cDNA using the Maxima H minus cDNA Synth Master Mix (Thermo Fisher Scientific, Basel, Switzerland) and random hexamers according to the manufacturer’s instructions. The thermal cycling conditions were as follows: 10 min at 25°C, followed by 15 min at 50°C and 5 min at 85°C (C1000 Thermal Cycler, Bio-Rad Laboratories, Hercules, CA; USA). The cDNA was diluted to a concentration of 5ng/µl with UltraPure^TM^DNase/RNase-Free distilled water (Thermo Fisher Scientific, Basel, Switzerland) and stored at −20°C until further use. Desalted oligonucleotide primers (Microsynth, Balgach, Switzerland) were used based on previous studies (Table 1). Cytokine profiling targeted a core panel capturing major axes of the immune response (Th1, Th2, inflammation, regulation), namely IFN-γ and TNF (indicative of Th1 and pro-inflammatory responses), IL-4 and IL-13 (associated with Th2-type responses), IL-6 (a pleiotropic cytokine involved in inflammation and acute-phase responses), and IL-10 (a key regulatory cytokine), chosen for their established roles in helminth infections. Additionally, the selection was informed by the availability and prior validation of reagents suitable for use in canine samples, as well as the technical feasibility of the assays in the context of an initial characterisation of PBMC responses to stage-specific antigens of *A. vasorum*.

**Table 1:** Primer sequences and annealing temperatures used for the quantitative PCR.

Gene	Primer sequence (5’−3’)	Annealing temperature (°C)	Reference
IL-4	F: CCAAAGAACACAAGCGATAAGGAA R: GTTTGCCATGCTGCTGAGGTT	61	[24]
IL-10	F: CCCGGGCTGAGAACCACGAC R: AAATGCGCTCTTCACCTGCTCCAC	63	[24]
IL-13	F: GAGGAGCTGGTCAACATCA R: TGCAGTCCGGAGACATTGA	60	[24, 25]
IFN-y	F: TCAACCCCTTCTCGCCACT R: GCTGCCTACTTGGTCCCTGA	61.1	[26]
TNF	F: CCCCGGGCTCCAGAAGGTG R: GCAGCAGGCAGAAGAGTGTGGTG	64	[24]
IL-6	F: CCCACCAGGAACGAAAGAGA R: CTTGTGGAGAGGGAGTTCATAGC	60	[27]
RPS5	F: TCACTGGTGAGAACCCCCT R: CCTGATTCACACGGCGTAG	62.5	[24, 25, 28]

*F* Forward, *R* Reverse

The ribosomal protein S5 (RPS5) encoding gene was used as reference gene. qPCR was based on the double-stranded DNA-binding dye SYBR green (Powerup SYBR Green Master Mix, Applied Biosystems by Thermo Fisher Scientific, Basel, Switzerland) using a Quantstudio 7 Flex Real Time PCR System (Applied Biosystems by Thermo Fisher Scientific, Basel, Switzerland). The primers had a final concentration of 1 µM each. For each reaction, 1 µl of cDNA template (5 ng) was used in a reaction volume of 10 µl. Reactions with primers with a Tm below 60°C started with 2 min at 50°C, 2 min at 95°C, followed by 40 cycles of 15 s at 95°C, 15 s at Tm of the primer used and 1 min at 72°C. This reaction was continued with 15 s at 60°C, followed by a gradual increase of 1.6°C every 15 s from 60 to 95°C to obtain a melt curve. For primers with a Tm above 60°C, the 40 cycles consisted of 15 s at 95°C and 1 min at Tm.

All test samples were run in triplicates and negative controls in duplicates. A no-RT control was included for each sample. Product melting curves revealed a product, and negative controls yielded negative results.

Amplification efficiencies for each primer pair were determined from a standard curve created by plotting the cycle threshold (Ct) values against the logarithm of the original template concentration. The slope of the standard curve was then calculated using linear regression analysis. Primer efficiencies (E) were determined using the following formula:

$$\:E=({10}^{\frac{-1}{slope}}-1)\times\:100$$

Primer efficiencies were between 90 and 120% for all primers. mRNA expression was normalised to the reference gene RPS5 and in comparison with the negative control. Relative gene expression was quantified using the efficiency-corrected 2^-ΔΔCT^ method and displayed as fold changes [29].

### Stimulation of PBMCs and flow cytometry

A total of 500,000 PBMCs were stimulated in RPMI medium supplemented with 10% foetal calf serum, 1 µg/ml cytosine-phosphate-guanine (CpG) and either 10 µg/ml of L1 antigen or ESP, or without antigen as control for three days at 37°C in a humidified atmosphere containing 5% CO₂. The cells were then processed for flow cytometry as described by Pantelyushin et al. [30] to assess potential changes in PBMC composition and activation/proliferation markers. Samples were acquired on a Cytek Aurora (Cytek Biosciences, Fremont, CA, United States). Data acquisition was performed using FlowJo v10.7 Software (BD Life Sciences). The gating strategy was performed as follows: total cells were first pre-gated to select singlets and live cells, followed by the gating steps illustrated in Fig. 5A, which follow the gating strategy described in Fig. 1 of Pantelyushin et al. [31]. First-stage larval antigen and ESP were selected because cytokine profiling revealed contrasting profiles that made them most informative for phenotypic analyses. While ESP elicited a regulatory/modest response, L1 induced a robust pro-inflammatory and proliferative signature. Cells were stimulated for 72 h, a time point chosen to balance early transcriptional events with later cellular phenotypes such as activation and proliferation.

**Fig. 1:**
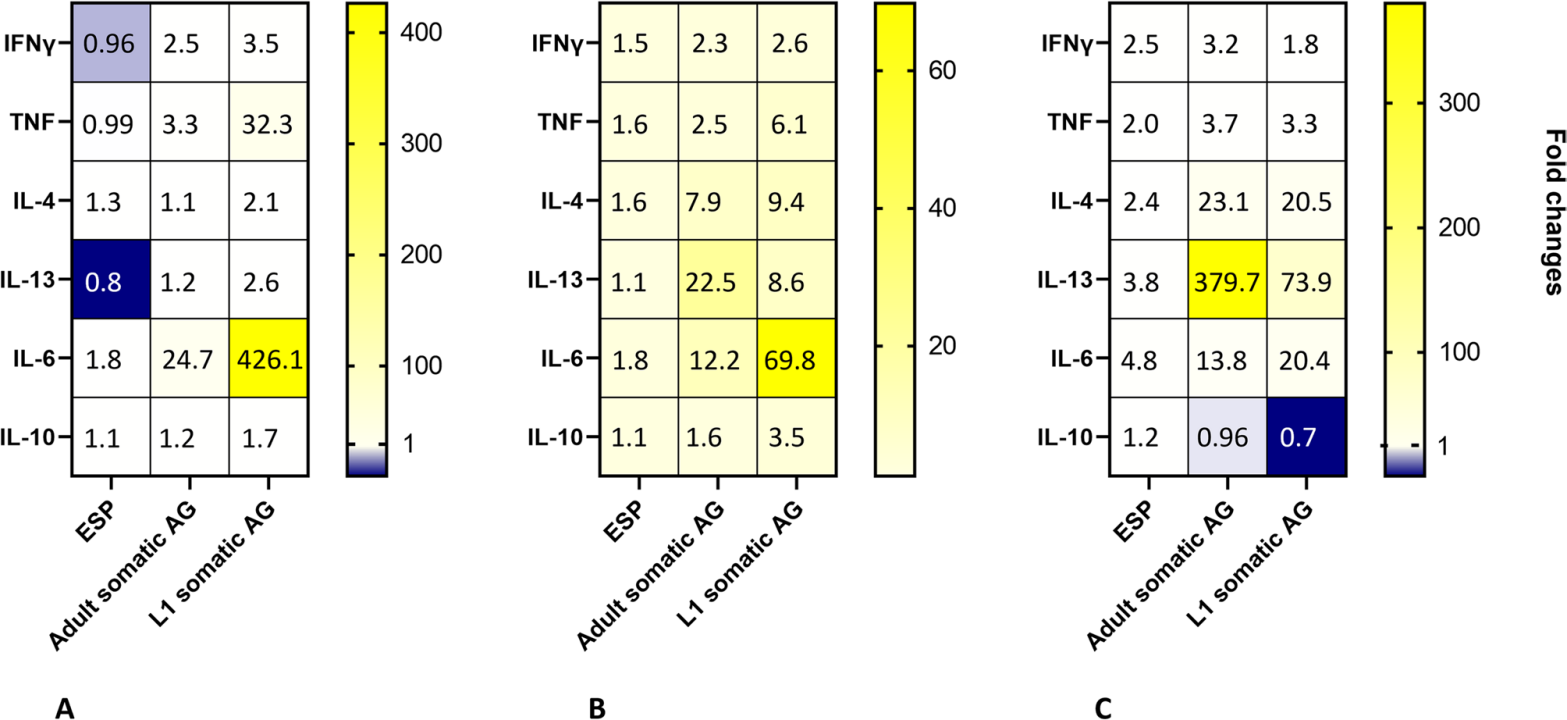
Cytokine gene expression in canine peripheral blood mononuclear cells (PBMCs) after exposure to *Angiostrongylus vasorum* antigens. Relative gene expression by PBMCs stimulated with *Angiostrongylus vasorum* excretory-secretory products (ESP), adult somatic antigen, or first-stage larval (L1) somatic antigen is displayed as fold change. Fold changes are indicated as numeric values within the cells of the heatmap. Each cell represents the mean of the corresponding gene across the replicates of seven individual donors. mRNA expression was normalised to the reference gene RPS5 and in comparison with the negative control

### Statistical analysis

Normality of the data was examined via density plots and the Shapiro-Wilk test. Linear mixed-effects models were fitted to assess differences across antigens, with individual dog ID as a random effect to account for repeated measures. Subsequently, relative gene expression across antigens was compared via estimated marginal means. Pairwise comparisons were adjusted for multiple testing using the Bonferroni method. Differences in relative gene expression across time points were assessed via linear mixed models with individual dog ID as a random effect as well as a two-way interaction term for time point and type of antigen. Estimated marginal means were used for post-hoc comparisons including the Bonferroni method to adjust p values. All analyses were carried out in R Software for Statistical Computing, version 4.3.2 [32]. Statistical significance was set at *p* ≤ 0.05 throughout the analyses.

Statistical analysis of the flow cytometry experiments was performed by one-way analysis of variance (ANOVA) with Bonferroni correction using GraphPad Prism version 10.0.0 for Windows (GraphPad Software, Boston, Massachusetts USA).

## Results

### Differential cytokine expression in canine PBMCs following stimulation with different antigens of *A. vasorum*

To assess the immune response to different antigens of *A. vasorum*, cytokine expression of canine PBMCs was assessed at various time points following stimulation with *A. vasorum* ESP, adult antigen, and L1 antigen. In response to stimulation with the three different *A. vasorum* antigens, canine PBMCs exhibited distinct patterns of cytokine upregulation and downregulation depending on the antigen across different time points. Relative gene expression is visualised in Fig. 1. Fold changes are compiled in Table 2.

At four hours, ESP induced only modest changes (Fig. 1; Table 2): IL-4, IL-6, and IL-10 were mildly upregulated, while IFNγ, TNF, and IL-13 were downregulated. In contrast, stimulation with the adult antigen and L1 antigen resulted in upregulation of all measured genes. Differences could be determined between stimulation with adult antigen and L1 antigen (*p* < 0.001) as well as between ESP and L1 antigen (*p* < 0.001) in the case of IL-6 and TNF. Furthermore, a difference existed for IL-10 between L1 antigen and ESP (*p* = 0.02).

At the 24-hour time point (Fig. 1; Table 2), all genes were upregulated following stimulation with ESP, although the upregulation was relatively moderate. Stimulation with adult and L1 antigen elicited a more pronounced upregulation of the examined genes. In the case of IL-4, differences in relative expression was present between L1 antigen and ESP (*p* = 0.01). This was also the case for IL-13 (*p* = 0.04). Differences between L1 antigen and ESP, as well as between L1 antigen and adult antigen, existed for IL-10 (*p* < 0.001 and *p* = 0.003, respectively), IL-6 (*p* < 0.001 and *p* = 0.002, respectively), and TNF (*p* < 0.001 and *p* = 0.003, respectively).

After 5 days (Fig. 1; Table 2), all genes remained upregulated following stimulation with ESP. Adult antigen induced an upregulation of IFNγ, TNF, IL-4, IL-6, and IL-13, whereas Il-10 was downregulated. A similar outcome was observed for stimulation with L1 antigen: while IL-10 was downregulated IFNγ, TNF, IL-4, IL-6, and IL-13 were all upregulated. Different relative expression was observed for IL-4 between L1 antigen and ESP (*p* = 0.01) as well as between adult antigen and ESP (*p* = 0.02). Moreover, a difference between L1 antigen and ESP was determined in the case of IL-10 (*p* = 0.01).

**Table 2:** Cytokine gene expression in canine peripheral blood mononuclear cells (PBMCs) after exposure to *Angiostrongylus vasorum* antigens

Gene	Fold change (stimulated vs. unstimulated)								
	ESP			Adult antigen			L1 antigen		
	4 h	24 h	5 days	4 h	24 h	5 days	4 h	24 h	5 days
IFNγ	0.96	1.5	2.5	2.5	2.3	3.2	3.5	2.6	1.8
TNF	0.99	1.6	2.0	3.3	2.5	3.8	32.3	6.1	3.3
IL-4	1.3	1.6	2.4	1.1	7.9	23.1	2.1	9.4	20.5
IL-13	0.8	1.1	3.8	1.2	22.5	379.7	2.6	8.6	73.9
IL-6	1.8	1.8	4.8	24.7	12.3	13.8	426.1	69.8	20.4
IL-10	1.1	1.1	1.2	1.2	1.6	0.97	1.7	3.5	0.7

Relative gene expression is represented as fold changes. Data are pooled from replicates of seven donor dogs. mRNA expression was normalised to the reference gene RPS5 and in comparison with the negative control. ESP: *Angiostrongylus vasorum* adult excretory-secretory products; L1: *Angiostrongylus vasorum* first-stage larvae

Relative gene expression varied across different time points following stimulation with *A. vasorum* antigens

**Fig. 2:**
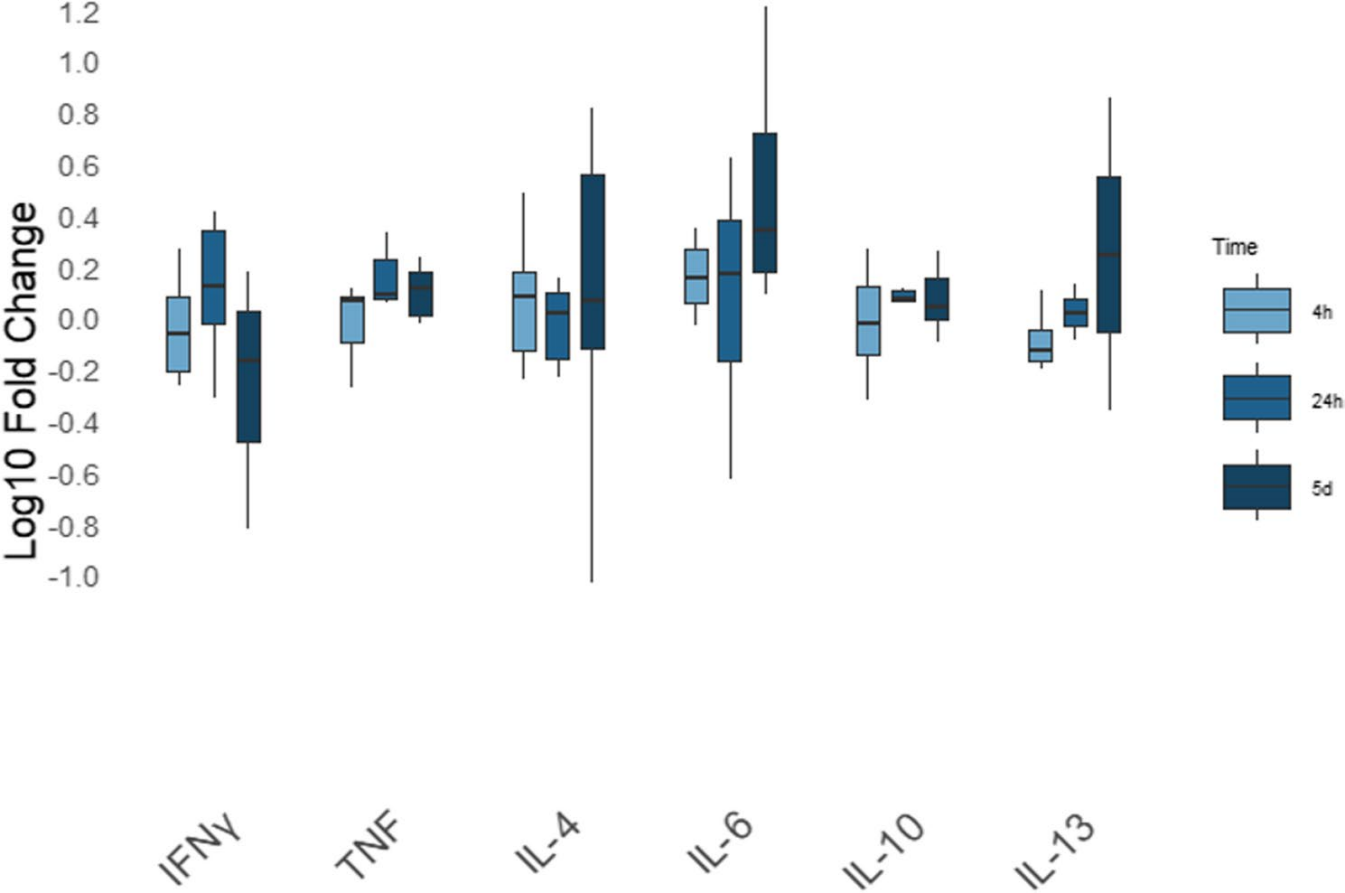
Differential gene expression across time points following stimulation with *Angiostrongylus vasorum* excretory-secretory products. Relative cyotokine gene expression by PBMCs after 4 h, 24 h and five days of stimulation with *Angiostrongylus vasorum* adult excretory-secretory products (ESP) across three different time points. Relative gene expression is shown as log₁₀-transformed fold changes. mRNA expression was normalised to the reference gene RPS5 and in comparison with the negative control. Box plots represent fold changes across the replicates of seven individual donors

**Fig. 3:**
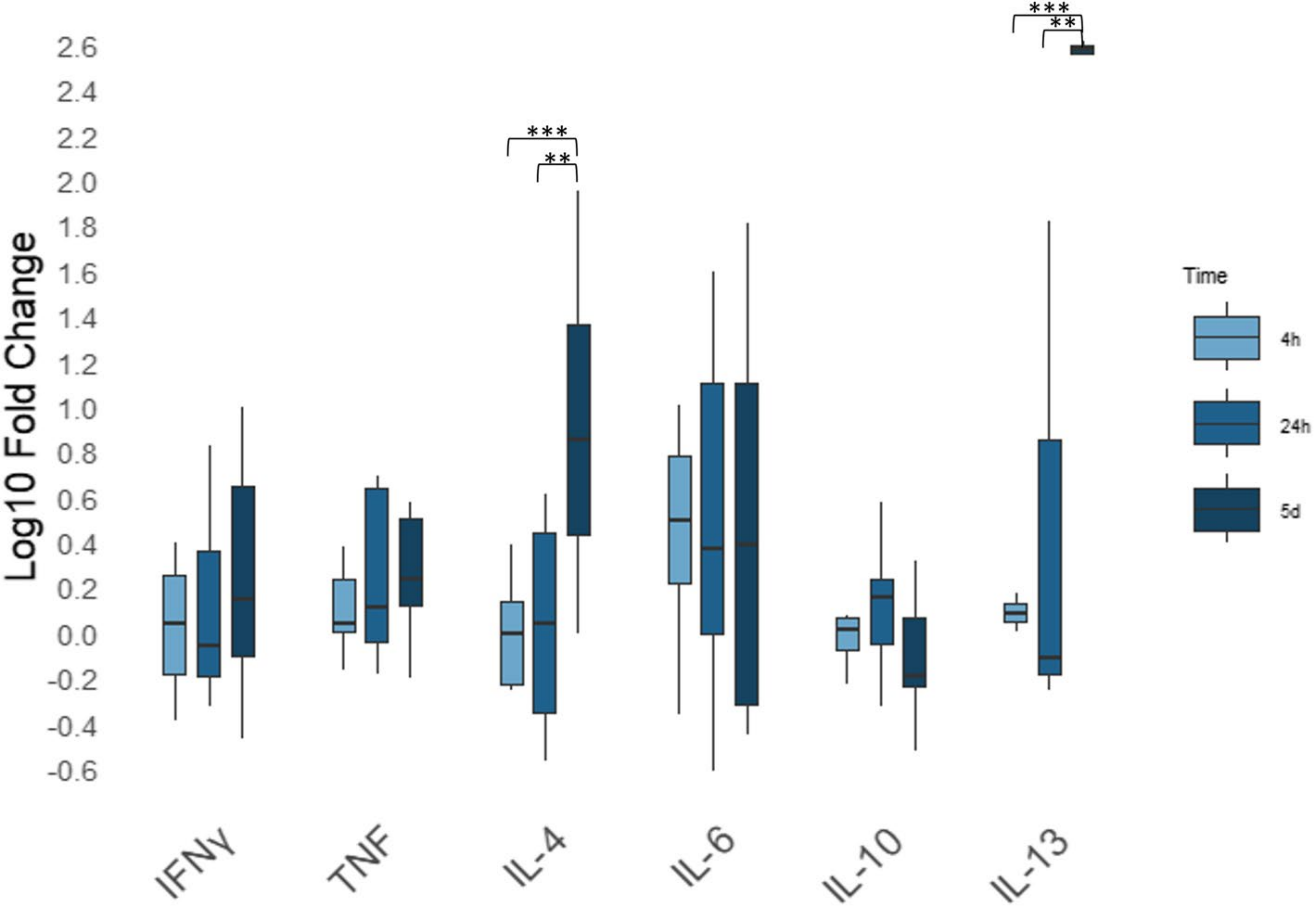
Differential gene expression across time points following stimulation with *Angiostrongylus vasorum* adult antigen. Relative cyotokine gene expression by PBMCs after 4 h, 24 h and five days of stimulation with *Angiostrongylus vasorum* adult antigen across three different time points. Relative gene expression is shown as log₁₀-transformed fold changes. mRNA expression was normalised to the reference gene RPS5 and in comparison with the negative control. Box plots represent fold changes across the replicates of seven individual donors. ** *p* ≤ 0.01; *** *p* ≤ 0.001

While no individual cytokine showed a statistically significant difference in relative expression at 4 h versus 24 h versus 5 days following ESP stimulation (Fig. 2, relative gene expression shown as log₁₀-transformed fold changes), adult antigen induced time-dependent changes in cytokine expression (Fig. 3, relative gene expression shown as log₁₀-transformed fold changes), characterised by clear upregulation of Th2-associated cytokines. Specifically, IL-13 increased from 1.2-fold at 4 h to 22.5-fold at 24 h and further to 379.7-fold at 5 days (*p* = 0.01 for 24 h vs. 5 d; *p* = 0.004 for 4 h vs. 5 d). IL-4 rose from 1.1-fold at 4 h to 7.9-fold at 24 h and reached 23.1-fold at 5 days (*p* = 0.01 for 24 h vs. 5 d; *p* = 0.001 for 4 h vs. 5 d).

**Fig. 4:**
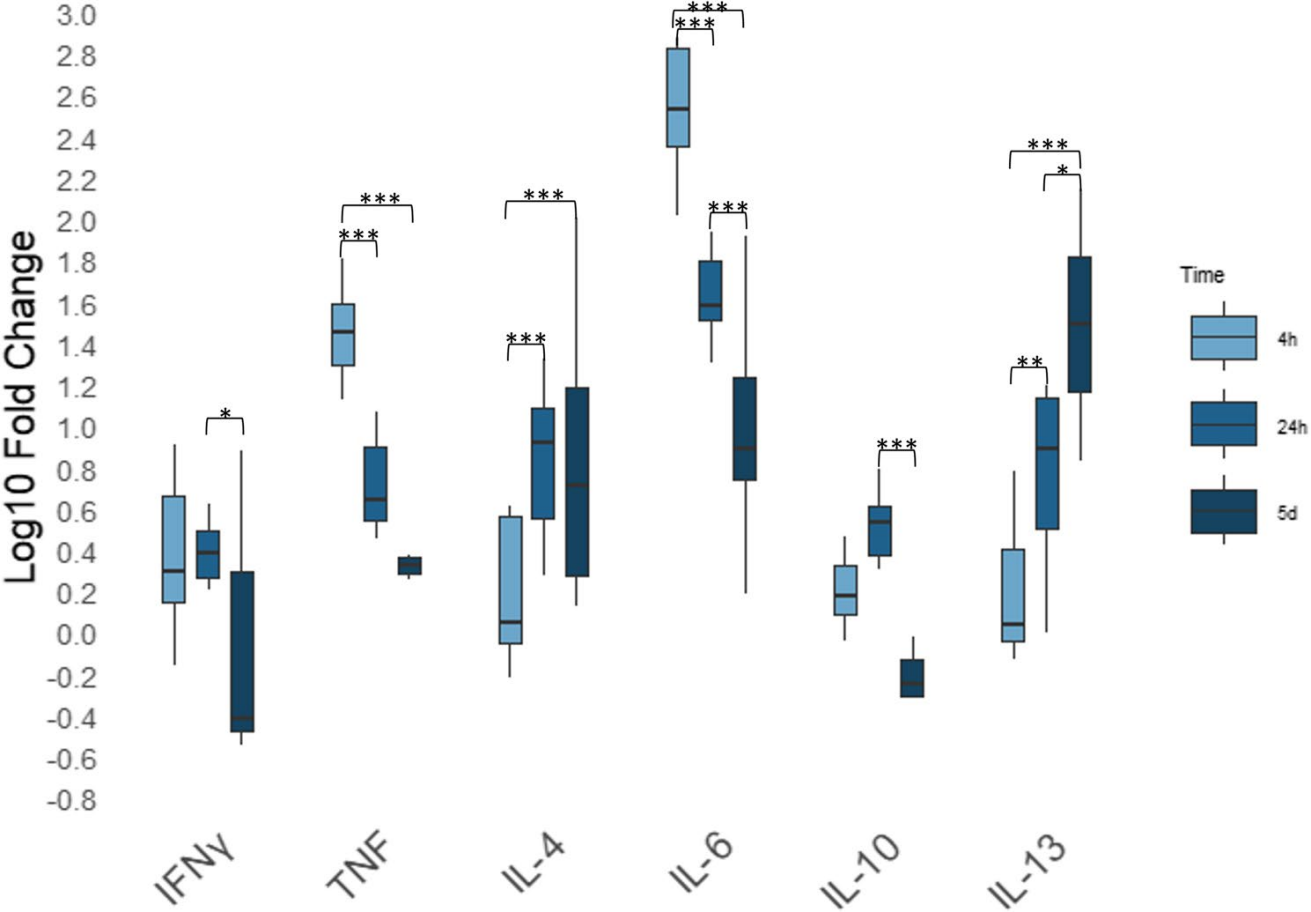
Differential gene expression across time points following stimulation with *Angiostrongylus vasorum* first-stage larval (L1) antigen. Relative cyotokine gene expression by PBMCs after 4 h, 24 h and five days of stimulation with *Angiostrongylus vasorum* L1 antigen across three different time points. Relative gene expression is shownas log₁₀-transformed fold changes). mRNA expression was normalised to the reference gene RPS5 and in comparison with the negative control. Box plots represent fold changes across the replicates of seven individual donors. * *p* ≤ 0.05; ** *p* ≤ 0.01; *** *p* ≤ 0.001

L1 antigen also induced clear increases and decreases over time (Fig. 4, relative gene expression shown as log₁₀-transformed fold changes). IFN-γ expression increased to 2.6-fold at 24 h and 1.8-fold at 5 days post-stimulation (*p* = 0.05). IL-10 was upregulated by 3.5-fold at 24 h following L1 stimulation, then decreased below baseline to 0.7-fold at 5 days (*p* < 0.001). IL-13 expression after stimulation with L1 stimulation rose from 2.6-fold at 4 h to 8.6-fold at 24 h and to 73.9-fold between 24 h and 5 days (*p* = 0.002 for 4 h vs. 24 h; *p* = 0.02 for 24 h vs. 5 d; *p* < 0.001 for 4 h vs. 5 d). IL-4 increased from 2.1-fold at 4 h to 9.4-fold at 24 h and 23.1-fold at 5 days (*p* = 0.001 for 4 h vs. 24 h; *p* < 0.001 for 4 h vs. 5 d). IL-6 expression after L1 stimulation peaked early at 426.1-fold at 4 hours, then was increased 69.8-fold after 24 h, and upregulated by 13.8-fold after 5 days (all comparisons *p* < 0.001). TNF was highest upregulated at 4 h (32.3-fold) following L1 exposure, then decreased to an upregulation of 6.1-fold at 24 hours and 3.3-fold after 5 days (*p* < 0.001 for 4 h vs. 24 h and 4 h vs. 5 d).

### Effect of antigenic stimulation on PBMC composition

To assess whether the observed cytokine differential expression induced changes in the composition of PBMCs, we performed a phenotypical characterisation by flow cytometry following a three-day stimulation with L1 antigen or ESP, comparing the results with unstimulated controls.

**Fig. 5:**
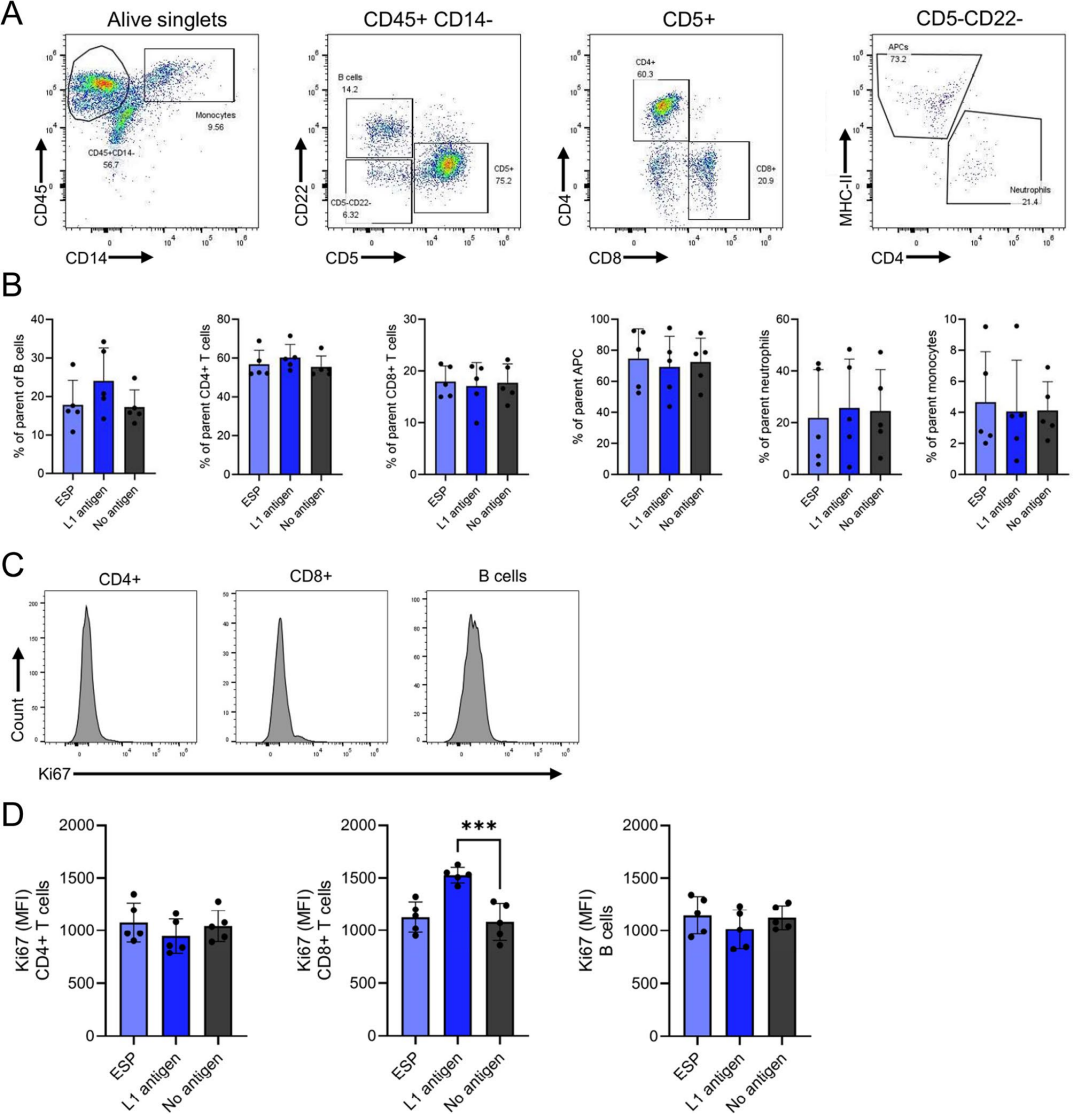
Immunophenotypical characterisation of canine peripheral blood mononuclear cells after stimulation with *Angiostrongylus vasorum* excretory-secretory products and first-stage larval (L1) antigen. **A** Gating strategy according to Pantelyushin et al. [30]. A representative sample is shown. **B** Frequency of parent population of CD4^+^ T cells, CD8^+^ T cells, B cells, monocytes, antigen presenting cells (APCs), and neutrophils. **C** Representative histogram of Ki67 expression levels. **D** Mean fluorescent intensity (gMFI) of Ki67 in CD4^+^ T cell, CD8^+^ T cell, and B cell subsets. Statistical significance of differences between groups was determined by one-way ANOVA with Bonferroni correction. *** *p* < 0.001

We then assessed major immune cell populations, including CD4⁺ and CD8⁺ T cells, B cells, monocytes, and CD14- CD22- APCs (Fig. 5A). These cell populations did not change in relative abundance upon stimulation with the different antigens (Fig. 5B). However, an increase in Ki67 expression (displayed as gMFI, Fig. 5C), a marker associated with cellular proliferation and active cell cycle progression, was detected in CD8⁺ T cells upon L1 antigen stimulation, suggesting enhanced proliferative activity in this subset (Fig. 5D). In contrast, Ki67 expression remained unchanged in CD4 + T cell and B cell populations upon L1 antigen stimulation and in any of the subsets stimulated with ESP.

## Discussion

The present work aimed at clarifying the dynamics of cytokine expression in canine PBMCs in response to different *A. vasorum* antigens, offering new insights into the host immune response to this parasite. Specifically, we explored both the dynamics of cytokine gene expression—comparing Th1, Th2, inflammatory, and regulatory profiles at 4 h, 24 h, and 5 days post-stimulation—and the downstream impact on PBMC composition and proliferative activity after a 72 h stimulation with ESP or L1 antigen. By integrating these complementary assays, we sought to capture a spectrum of early transcriptional events and later cellular outcomes elicited by stage-specific parasite antigens. Currently, the immune response to *A. vasorum* during infection is not well understood. In red foxes, evidence has suggested that there is no protective immunity [15, 33] and similar indications have been found in domestic dogs [14, 34, 35]. Even though recent serum proteomics analyses in both host species have corroborated the concept that the antiparasitic immune response differs between the two species [15, 36, 37], PBMC-mediated responses have not been further explored up to now.

ESP overall promoted a regulatory and Th2 cytokine profile. For example, at the four-hour time point, PBMCs exposed to ESP modestly upregulated IL-4, IL-6, and IL-10, whereas pro-inflammatory IFNγ, TNF, and IL-13 appeared to be downregulated, indicating a limited proinflammatory response. ESP components hence seem to modulate the immune response by promoting a balanced profile without substantial up- or downregulation of cytokines, with rather regulatory elements being favoured. This modulation is expected to create an environment conducive to the parasite’s survival, allowing it to evade detection and clearance by the host’s immune system. The ability of helminths to manipulate host immunity through their ESP has been well documented in various studies. For instance, Arora et al. [38] demonstrated that ESP of *Taenia solium* suppresses inflammatory pathways in human macrophages. Additionally, galectins secreted by *Brugia malayi* have been shown to polarise macrophages towards a phenotype of alternative activation and to selectively trigger apoptosis in Th1 cells, aligning with an overall immunological Th2 phenotype of chronic lymphatic filariasis infection [39].This highlights the strong ability of helminth ESP to interfere with effective host responses in favour of the parasite.

In contrast, stimulation with adult antigen elicited a broader cytokine profile with notable increases in IFNγ, TNF, and most prominently IL-6 expression. The extensive cytokine upregulation in response to adult antigen suggests that this antigen may trigger a more pronounced immune activation and inflammatory response. The substantial upregulation of IL-6 — a key cytokine in acute-phase responses and inflammation pathways [40–42] — suggests that certain antigenic components of adult *A. vasorum* stages can elicit a strong immune response in the host. The strong increase in TNF and IFNγ further corroborates this finding, as these cytokines are hallmarks of Th1 responses and contribute to the activation of immune effector cells and the amplification of inflammation [43, 44]. However, in vivo, the host immune system may not encounter the full spectrum of adult somatic antigens due to either the parasite’s intactness or its ability to sequester or limit antigen exposure, potentially dampening strong immune reactions. For example, the liver flukes *Fasciola hepatica* and *Fasciola gigantica* can alter their surface antigens and express the leukocyte marker CD77 in their tegument. This strategy reflects molecular mimicry, enabling them to evade the host immune system and maintain infection [45, 46]. In this very context, future work could prioritise purified cuticular antigens of *A. vasorum* to determine whether surface-exposed molecules mediate similar mimicry or immune masking, and to assess whether these antigens could elicit stronger or more protective responses when used *in vitro* or in vaccine formulations. Molecular mimicry may partly explain why infected dogs are not able to successfully expel the parasite as their immune responses are not fully activated against it. On the other hand, in cases where parasites appear to perish within the host and undergo degradation, antigens that were previously not exposed to the host system, i.e. hidden antigens, may be released in abundance, prompting strong and immediate reactions within the host organism, potentially contributing to the varied, sometimes severe clinical manifestations seen in canine angiostrongylosis. For example, inflammatory cell counts and exacerbated clinical signs have been observed after anthelmintic treatment in dogs experimentally infected with *A. vasorum*. Specifically, 12 days after treatment transient and mild to moderate neutrophilic leucocytosis and a mild increase in lymphocyte count were present, indicating an enhanced inflammatory response and a cellular immune reaction to dead parasites [11]. However, this may only represent one aspect of severe clinical manifestations in *A. vasorum*-infected dogs and the overall severity of clinical signs may rather arise from a combination of factors such as parasite burden, chronicity, tissue migration by L1, vascular damage, immune-mediated pathology, and host-coagulopathies [11–13, 37, 47–49].

L1 antigen resulted in the most substantial upregulation across all cytokines examined. At the four hours time point, L1 antigen appeared to be particularly effective at stimulating a strong pro-inflammatory immune response, represented by an extraordinary upregulation of IL-6. This aligns well with pathological findings in the lungs of *A. vasorum*-infected dogs, where L1 cause extensive damage, including excessive presence of cellular infiltrates of mononuclear cells [11]. The evident differences in the cytokine response to L1 antigen vs. both ESP and adult antigens further underscore the strong potential of L1 to trigger a pronounced pro-inflammatory reaction. The pro-inflammatory response is accompanied at the cellular level by an increased expression of Ki67 in CD8 + T cells. At the 24-hour time point, L1 antigen demonstrated the highest levels of relative gene expression across the three tested antigens. The increased expression of IL-4 and IL-13, indicates a shift towards a Th2 cytokine profile which may facilitate parasite survival via immune tolerance [20]. In this very context, the differences observed in IL-4 and IL-13 expression between L1 antigen and ESP may suggest that L1 antigen could be particularly effective at skewing the immune response towards a more regulatory Th2 profile. At the five-day time point, L1 antigen continued to provoke a pronounced upregulation of Th2 cytokines IL-4 and IL-13, aligning with a shift towards a modified immunological profile.

Interestingly, L1 antigen emerged as a strong driver of a Th1 cytokine profile in canine PBMCs, which is a central finding of this study. This result is particularly striking given the expectation that parasites typically induce a Th2 cytokine profile [1, 6, 7]. The robust Th1 cytokine profile by the L1 antigen suggests that certain structural components or motifs within the antigen may be particularly immunogenic, potentially promoting the activation of Th1-associated cytokines such as IFN-γ and TNF. The strong inflammatory capacity of this stage of *A. vasorum* is consistent with the tremendous damage these larvae cause in infected lung tissue [11]. However, in natural infections, clinical outcomes range from subclinical infections to severe granulomatous pneumonia with extensive infiltration of the lung tissue with inflammatory cells, vascular damages, and fibrosis [11, 47, 49, 50]. Subclinical infections may reflect are more balanced immune response or low parasite burden, where regulatory mechanisms limit excessive pathology. By contrast, high larval loads or a dysregulated, more extreme response, may shift pathogenesis towards inflammation.

Moreover, our *in vitro* data revealed a temporal sequence in which L1 antigen elicited an early pro-inflammatory/Th1-like surge followed by a progressive Th2/regulatory profile: for example, IL-4 and IL-13 expression increased progressively over 24 h to 5 days, even if some cytokines (e.g., IFN-γ or IL-10) may transiently decline after an initial peak. This biphasic pattern may reflect the concepts of upregulation of pro-inflammatory mediators and subsequent immune tolerance mechanisms for the same antigen. In vivo, additional factors that come into play, such as interactions with tissue-resident cells, chemokine gradients, complement, and host-genetic variation may shape the inflammatory milieu beyond what the culture system of the present study can capture. Thus, the results from the present study illustrate the potential of L1 antigen to drive both pro-inflammatory and Th2 pathways. The findings raise intriguing questions about which molecular features of L1 antigens trigger each phase and how their recognition by host pattern recognition receptors or memory T-cell repertoires influences the switch from initial Th1 activation to later Th2/regulatory profile.

We observed a marked increase in Ki67 expression in CD8⁺ T cells following L1 antigen stimulation without a corresponding rise in their overall frequency among PBMCs. This pattern may indicate activation and proliferation of pre-existing memory CD8⁺ T cells rather than a net expansion of the CD8⁺ population. In other words, Ki67 upregulation shows that these cells have entered the cell cycle and are dividing, but factors such as concurrent cell death, homeostatic mechanisms in culture, or proliferation of other subsets could maintain the overall proportion of CD8⁺ cells at a steady level. A true net expansion would manifest as an increased percentage or absolute count of CD8⁺ T cells relative to other PBMC subsets, which we did not detect. It is also important to note that although Ki67 expression was significantly increased in CD8 + T cells upon L1 antigen stimulation, the magnitude of this change may not be sufficient to drive a phenotypic shift or a noticeable increase in CD8 + T cell abundance at the population level, especially if it is limited to a fraction of the cells or balanced by apoptosis.

In contrast, Ki67 expression remained unchanged in CD4⁺ T cell and B cell populations upon L1 antigen stimulation, and no subset exhibited altered proliferation after ESP stimulation, indicating a selective proliferative response in CD8⁺ T cells. While Th1/Th2 terminology commonly refers to CD4⁺ helper subsets [51–53], CD8⁺ T cells can also produce IFN-γ and participate in a type 1 cytokine milieu [54, 55]. Their proliferation under L1 stimulation hence may align with the pro-inflammatory environment rather than expansion of a CD4⁺ Th1 subset. Because relative frequencies rather than absolute cell numbers were measured, proliferative activity in CD8⁺ cells may be masked if total cell counts change or if proliferating cells undergo apoptosis. Furthermore, the in vitro culture lacks in vivo survival and trafficking signals that could support accumulation of cells. This selective activation may reflect a higher precursor frequency or stronger co-stimulatory engagement for CD8⁺ memory cells in response to L1 antigen, whereas CD4⁺ T cells and B cells might require different signals or longer timeframes to proliferate.

Cytokine gene expression was assessed at early (4 h), intermediate (24 h) and late (5 days) time points to capture the kinetics of transcriptional responses, ranging from immediate signaling to longer-term shifts. Flow cytometry, by contrast, was performed after a 72 h stimulation to allow sufficient time for phenotypic activation and proliferative markers to manifest in PBMC subsets as transcriptional activation may precede observable changes in cell-surface or intracellular markers [56, 57]. Moreover, gene expression was measured for all antigens at 4 h, 24 h, and 5 days, while flow cytometry was conducted only for L1 antigen and ESP. L1 antigen and ESP were chosen because they elicited the most divergent cytokine responses, making them most informative for potentially contrasting phenotypes. While direct quantitative correlation between mRNA levels at specific time points and phenotypic read-outs at 72 h may not be possible, the two assays provide complementary insights into the sequential stages of the immune response. Despite the narrower antigen selection for flow cytometry, analysing L1 and ESP remains valid as these antigens represent the extremes of the immune response spectrum observed in gene expression, thereby yielding meaningful contrasts in phenotypic activation and proliferation. The early transcriptional surge in pro-inflammatory cytokines after L1 stimulation could drive the downstream cellular response captured at 72 h, exemplified by increased Ki67 expression in CD8⁺ T cells. Conversely, the modest, largely regulatory cytokine profile elicited by ESP is mirrored by minimal changes in proliferation or subset composition at the same phenotypic time point. Hence, these differing intervals reflect complementary aspects of the immune response. Although the conditions are not directly comparable, this approach enabled us to link dynamic gene expression patterns with downstream functional outcomes, recognising that transcriptional peaks may occur before or beyond the timeframe in which phenotypic changes are most readily observed.

Even though monocytes and other antigen-presenting mononuclear cells were included in the flow cytometry panel, their relative frequencies did not change after 72 h stimulation with either L1 antigen or ESP. This lack of shift in proportions may not necessarily imply absence of activation: monocytes and APCs may respond functionally by upregulation of co-stimulatory molecules or secretion of cytokines without proliferating or altering overall subset frequencies *in vitro* [58]. Since we did not measure activation markers (such as CD80, CD86 or MHC II upregulation) on these cells, it remains possible that antigen exposure induced phenotypic or functional changes not reflected in cell abundance. Furthermore, the culture conditions and antigen concentrations used may favour rapid cytokine release by monocytes without driving their expansion or survival changes detectable by Ki67 or frequency analysis. In vivo, monocytes and tissue-resident APCs likely encounter antigen in a more complex microenvironment with additional signals that can modulate recruitment, differentiation, or proliferation, whereas such signals are absent in a simplified *in vitro* culture system. Overall, these points highlight that further investigations into functional activation markers and cytokine production by monocytes and APCs are required to clarify their contribution in the host-parasite interaction during canine angiostrongylosis.

The differences in relative expression of cytokines across time points not only underscore the complexity of host-helminth interactions but also indicate that the timing and nature of antigen exposure may well be critical factors influencing the immunological profile and, by extension, the clinical picture observed in canine angiostrongylosis [1, 4, 20]. This is particularly reflected by the pronounced cytokine response elicited by adult somatic and L1 antigen, which, however, does not confer protective immunity in vivo, nor enable clearance of the parasite. Such early responses could set the stage for later complications even if inflammation is subsequently modulated. Early pro-inflammatory peaks (e.g., IFN-γ, TNF, IL-6) may drive vascular inflammation and tissue damage, contributing to severe clinical signs such as bleeding disorders, where endothelial injury and dysregulated coagulation cascades are involved [19, 22, 36, 37, 59]. Moreover, such responses may contribute to individual variation in the clinical presentation of canine angiostrongylosis, particularly in relation to severe signs such as bleeding disorders [12, 31, 48] or verminous pneumonia [11, 60]. Subsequent or concurrent Th2/regulatory shifts (e.g., IL-4, IL-13, IL-10) may promote immune tolerance that may limit overt inflammation but also permit parasite persistence.

However, a strong Th2 milieu can have pathological implications. IL-4/IL-13 drive eosinophil recruitment and activation, which may cause tissue damage and fibrosis in the lungs, and may influence vascular permeability and remodeling [61]. Additionally, regulatory dampening of protective responses may allow parasite burden to increase, exacerbating pathology. This regulatory milieu could also influence haemostatic balance by modulating endothelial and macrophage functions, potentially exacerbating coagulopathies under certain conditions [61–64]. This is further corroborated by the clear trend towards a Th2 response over time, with increased expression of the corresponding cytokines at later time points. Thus, while Th2/regulatory responses can limit acute inflammation, they may inadvertently facilitate pathogenesis and pathological changes by allowing persistent or high-level infection, promoting eosinophilic and fibrotic pathology, and by insufficiently controlling early vascular injury [61]. Such a chronic, regulated immune response may translate into limited acute damage but sustained vascular alterations, aligning with clinical observations where some dogs exhibit severe inflammatory pathology and bleeding, while others manifest a more indolent, chronic progression. Overall, the interplay between early pro-inflammatory peaks and later Th2/regulatory responses may help explain why some dogs develop severe clinical signs (potentially also dependent on worm burden, chronicity, or further ailments) whereas others experience a more chronic progression of the infection [11, 37, 47, 50].

## Conclusions

The results of this study provide insights into the variability of cytokine responses induced by three different stages of *A. vasorum* in canine PBMCs. The diverse and sometimes contrasting cytokine responses ranging from an early, robust Th1/pro-inflammatory signature (including CD8⁺ T-cell proliferative activity) to a later-emerging shift toward Th2/regulatory responses, underscore the complex, stage-specific host interaction with this parasite. Such temporal and antigen-dependent shifts may contribute both to the inflammatory pathology seen (e.g. lung tissue damage due to L1 migration driven by Th1-type mediators and CD8⁺ activation) and to the establishment of a regulated, permissive environment favouring chronic infection (via Th2/regulatory elements). While Th2/regulatory elements can limit acute inflammation, they may also allow higher parasite burdens to persist and promote eosinophilic or fibrotic pathology, thereby contributing to severe or even fatal disease under particular conditions. Further investigation into the antigenic components driving this Type 1 polarisation could offer valuable insights into the immune evasion strategies of *A. vasorum*, the mechanisms of pulmonary pathogenesis, and the parasite’s capacity to establish chronic infections. Interventions aiming to enhance protective immunity must balance enhancing effective anti-parasitic responses while minimising immunopathology. For example, identifying stage-specific antigens that preferentially elicit protective Th1 responses could inform vaccine design, and cytokine signatures may serve as biomarkers or guide adjunct immunomodulatory therapies to mitigate severe outcomes. Further research dissecting the molecular drivers of these stage-specific responses may guide therapies that selectively bolster protective mechanisms or attenuate harmful inflammation, thereby improving clinical outcomes and prevention strategies in canine angiostrongylosis.

## Data Availability

The datasets used and/or analysed during the current study are available from the corresponding author on reasonable request.

## Abbreviations

APC: Antigen-presenting cell
ESP: Excretory-secretory products
IFN: Interferon
IL: Interleukin
L1: First-stage larvae
PBMCs: Peripheral blood mononuclear cells
PBS: Phosphate-buffered saline
qPCR: Quantitative polymerase chain reaction
NTC: No-template control
RT: Reverse transcription, reverse transcriptase
TNF: Tumor necrosis factor
CpG: Cytosine-phosphate-guanine
